# Changing patterns of malaria during 1996-2010 in an area of moderate transmission in Southern Senegal

**DOI:** 10.1186/1475-2875-10-203

**Published:** 2011-07-25

**Authors:** Philippe Brasseur, Malick Badiane, Moustafa Cisse, Patrice Agnamey, Michel T Vaillant, Piero L Olliaro

**Affiliations:** 1 Institut de Recherche pour le Développement (IRD), UMR 198, Rue Wagane Diouf × Georges Pompidou, Dakar, Sénégal; 2 District Médical d'Oussouye, Oussouye, Sénégal; 3 Programme National de Lutte contre le Paludisme (PNLP), Ministère de la Santé et de la Prévention, Rue Aimé Césaire, Dakar, BP. 4024, Sénégal; 4 Laboratoire de Parasitologie-Mycologie, Centre Hospitalier Universitaire, Place Victor Pauchet, Amiens, 80054, France; 5 Methodology and Statistical Unit, Center for Health Studies, CRP Santé, 1a Rue Thomas Edison, Strassen, L-1445, Luxembourg; 6 Unité 3677, Bases thérapeutiques des inflammations et infections, Université Victor Segalen Bordeaux 2, 145 Rue Leo Saignat, Bordeaux, 33000, France; 7 UNICEF/UNDP/WB/WHO Special Programme for Research & Training in Tropical Diseases (TDR), 20 avenue Appia, CH-1211 Geneva 27, Switzerland; 8 Centre for Tropical Medicine and Vaccinology, Nuffield Department of Medicine, University of Oxford, Churchill Hospital, Oxford, OX3, UK

## Abstract

**Background:**

Malaria is reportedly receding in different epidemiological settings, but local long-term surveys are limited. At Mlomp dispensary in south-western Senegal, an area of moderate malaria transmission, year-round, clinically-suspected malaria was treated with monotherapy as per WHO and national policy in the 1990s. Since 2000, there has been a staggered deployment of artesunate-amodiaquine after parasitological confirmation; this was adopted nationally in 2006.

**Methods:**

Data were extracted from clinic registers for the period between January 1996 and December 2010, analysed and modelled.

**Results:**

Over the 15-year study period, the risk of malaria decreased about 32-times (from 0.4 to 0.012 episodes person-year), while anti-malarial treatments decreased 13-times (from 0.9 to 0.07 treatments person-year) and consultations for fever decreased 3-times (from 1.8 to 0.6 visits person-year). This was paralleled by changes in the age profile of malaria patients so that the risk of malaria is now almost uniformly distributed throughout life, while in the past malaria used to concern more children below 16 years of age.

**Conclusions:**

This study provides direct evidence of malaria risk receding between 1996-2010 and becoming equal throughout life where transmission used to be moderate. Infection rates are no longer enough to sustain immunity. Temporally, this coincides with deploying artemisinin combinations on parasitological confirmation, but other contributing causes are unclear.

## Background

The Global Malaria Action Plan [1] aims at (i) providing universal coverage (prevention plus case management) by 2010; (ii) reducing the malaria burden and deaths by 50% in 2010, 75% in 2015 vs. 2000, (iii) eliminating malaria in 8-10 countries by 2015; and (iv) eradicating malaria in the long-term.

The foundations for this plan are that these objectives can be achieved (as locally applicable) by making available and deploying at the same time interventions, which have proved beneficial individually, including insecticide-treated nets (ITN), insecticide residual spraying (IRS), and artemisinin-based combination therapy (ACT).

While the Malaria Action Plan was being launched, a Lancet commentary [2] found that, in the previous 10 years, some 80 papers had reported trends in malaria incidence (increased in 15%, not changed in 14% and decreased in 71%). The same Lancet issue published two papers reporting decreasing malaria burden in the Gambia and Kenya [3,4]. These general trends towards a reduction of malaria were confirmed by the World Health Organization (WHO) in the Malaria Report 2009 [5] and 2010 [6] and other papers [7,8].

Against this scenario, a central question is now to collect the information which will allow properly documenting and interpreting these trends across endemic areas, while being cognisant that long-term trends are notoriously difficult to interpret (likely to be multi-factorial) and prone to bias (reporting and publication biases).

This was a facility-based study of malaria risk over a period of 15 years during which malaria treatment policy changed from administering anti-malarial monotherapy on clinical grounds to artemisinin combination on parasitological confirmation.

## Methods

### Study area

Mlomp (between latitudes 12°36' and 12°32'N and longitudes 16°33' and 16°37'E) is a village in the district of Oussouye, south-western Senegal. Since 2000, the population has been stable with about 7,600 inhabitants [9,10] and was about 6,000 before [10]. The village has a dispensary - a government peripheral health centre reporting to the district medical officer in Oussouye; the dispensary has one nurse, one technician and three health workers and is run by a congregation. No private pharmacies or drug sellers exist in the village.

Here, malaria is reportedly mesoendemic; transmission of moderate intensity occurs year-round with a seasonal peak during the rainy season (roughly July-December). Updated information on transmission intensity and entomological inoculation rates (EIR) is not available (the latest study done in the late 1990's report EIR of ~25 infected bites per person per year [11].

The malaria treatment policy in Senegal has been chloroquine or quinine administered after clinical presumptive diagnosis until 2004. Following an interim policy of amodiaquine plus sulphadoxine/pyrimethamine (SP), artesunate-amodiaquine (AS+AQ) became the first-line treatment in 2006 and rapid diagnostic tests (RDT) were made available from 2007 for the diagnosis of malaria. After a clinical trial in 1999 [12], AS+AQ was initially used for children under 10 in the rainy season and then extended to the whole population [13-15]. The implementation of the new policy (artesunate-amodiaquine after parasitological confirmation) and the previous practice (quinine or another drug on clinical grounds) have coexisted during this period. The efficacy and safety of artesunate-amodiaquine have been documented [13,15].

The age structure of the malaria cases in this area remained stable over several years [15] and consistent with other areas of similar endemicity in Senegal [16], with the highest risk of malaria being between 6-15 years (57% of cases), that extends also later in life (8% were 21-30, 7% 30 and above).

### Data

Data on overall consultations for fever, malaria treatments, parasitological test results and patients' age were extracted from the clinic registers of the dispensary of the village of Mlomp for the period of 01/01/1996 to 31/12/2010.

Information on patient demography (age as date of birth from the patient's identity card or the health card if a child, and sex), clinical signs (measured axillary fever) and reasons for consulting, diagnosis and treatment are recorded on a standard registry delivered by the Ministry of Health. Registries are kept by the nurse and filled daily, and inspected a monthly basis by the district medical officer.

Parasitological diagnosis was by thick (counting 200 WBCs) and thin blood smear before 2007, and by HRP-2 based RDT from 2007 (quality-controlled systematically by blood smear at the dispensary).

The rate of positive parasitological tests was applied to the overall number of treatments to obtain the projected malaria cases. The rates of treatments given after a positive, negative or no parasitological test were calculated out of the number of treatments and the number of consultations for fever. The number of AS+AQ treatments was also divided by the number of treatments to give the relevant rate.

Age (in months or years) was analysed both as a continuous variable and by age categories (0-5 years, 6-10, 11-15, 16-20, 21-30 and > 30). The distribution of age throughout the years between patients with positive and negative malaria tests was assessed using a general linear model. The parasitological tests were either microscopy (stained thin plus thick smear) or a RDT based on histidine-rich protein II (HRP-II).

Counts of malaria treatments were reported per year on aggregate and also specifically for each of the treatments with quinine, AS+AQ, chloroquine and AQ+SP.

### Age-dependent risk of malaria

Counts of positive parasitological tests were reported and compared over the years for the age categories 0-5y, 6-10y, 11-15y, 12-15y, 16-20y, 21-30y and > 30y.

### Parasitological tests count model

A count data model was used to evaluate the effect of parasitological test results (positive or negative), age and year on the count of parasitological tests done in Mlomp. Count data were tested for over-dispersion using the Lagrange multiplier test. The Poisson model was used when no over-dispersion was found, otherwise the negative binomial model was used. The model accounted for patient's age, year, interaction between age and year (the effect of age nested into the year), parasitological test result, its interaction with year and its interaction with age and year. Age was first introduced in the model as a continuous and subsequently as a categorical variable with the group 0-5 years as the reference.

The relative risk (RR) of a lower or higher count of parasitological tests with a 95% confidence interval (95%CI) was assessed with the age category 0-5y, the year 1996 and a negative parasitological test as the references of the corresponding variables.

### Malaria risk model

The odds of having parasitologically-confirmed malaria were evaluated over the years by using a logistic model for a positive parasitological test (i.e. positive vs. negative parasitological test as dependent variable), firstly with the year and age considered independently, and then with the interaction between them. The Odds Ratio (OR) of a parasitological test outcome depending on age were assessed for each year. The addition of a quadratic term of age (age*age) in the model was not significant, therefore, a linear relationship between age (continuous) and the probability of positive parasitological test was used.

Since (i) there was a significant interaction between age and year in the model, and (ii) patients data are independent from year to year, single models for each year including age as continuous variable were used to estimate the OR of malaria.

## Results

### Malaria burden in 1996-2010

#### Overall trends

Between January 1996 and December 2010, a total of 105,417 patients consulted for fever and 45,907 (44%) received an anti-malarial treatment (whether on clinical or parasitological confirmation); 30,685 (29% of all consultations for fever) underwent parasitological confirmation (either thin/thick smear or RDT) and 27,081 had also age recorded (26%); 10,786 (24%) tested positive for *Plasmodium falciparum*. The projected number of malaria cases over the entire period was 18,528.

**Figure 1:**
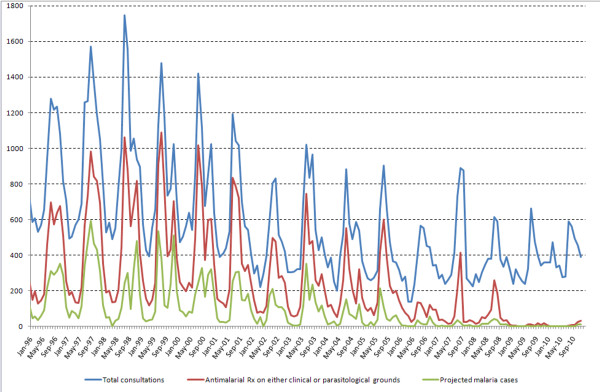
**Monthly records of consultations for fever, anti-malarial treatments dispensed (on either clinical or parasitological grounds) and projected real (parasitologically-confirmed) malaria cases in Mlomp during 1996-2010**.

Over time, all these figures decreased steadily. Table 1 presents the yearly and Figure 1 the monthly records of consultations for fever, anti-malarial treatments dispensed and projected real (parasitologically-confirmed) malaria cases during 1996-2010.

**Table 1:** Yearly records of consultations for fever, anti-malarial treatments dispensed (on either clinical or parasitological grounds) and projected real (parasitologically-confirmed) malaria cases in Mlomp during 1996-2009

Year	*Consultations for fever*	*Anti-malarial treatments*	*Projected Malaria Cases*	*Parasitologically Tested*	*Parasitological test result and age recorded*	*(% positive)*
**1996**	10270	4637	2237	2148	2129	48.2%
**1997**	11274	5743	3065	2379	2376	53.4%
**1998**	11006	5680	1857	2275	2221	32.7%
**1999**	9807	5635	2243	2414	2372	39.8%
**2000**	9361	5391	2474	3466	2488	45.9%
**2001**	7910	4432	2260	3362	2524	51.0%
**2002**	5596	2834	968	2259	2047	34.1%
**2003**	6040	2948	1212	3192	2707	41.1%
**2004**	6068	2781	870	2099	1799	31.3%
**2005**	5212	2673	724	2139	1853	27.1%
**2006**	4131	1092	254	1681	1343	23.2%
**2007**	5128	996	125	1628	1535	12.6%
**2008**	4485	885	201	850	848	22.8%
**2009**	4213	93	18	464	462	19.7%
**2010**	4916	87	20	381	377	23.1%

**All**	105417	45907	18528	30737	27081	37.5%

The table presents also the total number of parasitological tests done each year and their outcome.

RDT introduced in 2007.

**Figure 2:**
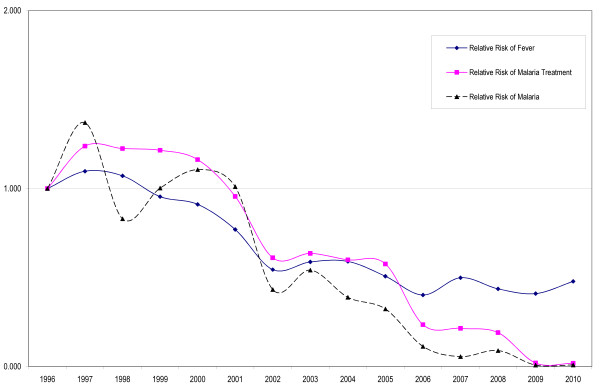
**Relative Risk (RR) of consulting for fever, receiving a malaria treatment and having a parasitologically-proven malaria attack expressed in person-year relative to 1996**.

As an example, when comparing the years before the start of the new malaria treatment practice (1996-1999) to the period 2007-2010, the calculated risk of malaria decreased approximately 32 times from ~0.4 to ~0.012 episodes person-year. Concomitantly, consultations for fever decreased 3-times (from ~1.8 to 0.6 visits person-year) and anti-malarial treatments decreased 13-times (from ~0.9 to 0.07 treatments person-year) (Figure 2).

#### Malaria treatments and treatment practices

**Figure 3:**
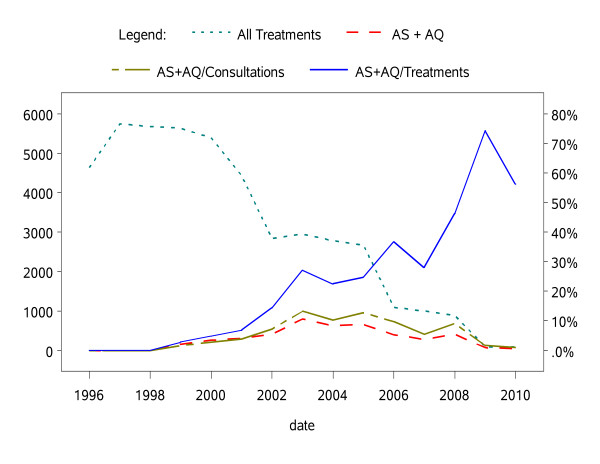
**Anti-malarial treatments dispensed at the Mlomp out-patient clinic**.

The number of malaria treatments was > 4,400/year between 1996-2001 (peak in 1998 with 5,680 treatments) and then started to decline steadily from 2002 (Table 2 and Figure 3). Four drugs (quinine 82%, AS+AQ 9%, chloroquine 6%, and amodiaquine + SP 1%) accounted for ~98% of all drugs used on either clinical or parasitological grounds over the 15-year period. Overall, 4,424 AS+AQ and 37,669 quinine treatments were delivered from 1996 to 2009. Quinine use was high (~80-95%) until 2003, then steadily decreasing (but was still accounting for 38 of the 87 treatments in 2010); chloroquine is no longer used since 2007; amodiaquine+SP was an interim recommendation of the national malaria control programme (used here only in 2005-2006); AS+AQ is the current policy in Senegal and its use has been steadily extending (69/93 and 49/87 treatments in 2009 and 2010). In absolute terms, the number of treatments is decreasing sharply.

**Table 2:** Anti-malarial treatments provided

YEAR	All Treatments	AS + AQ	Q	CQ	AQ + SP	Other Treatments
**1996**	4637	0	4132	504	0	1
**1997**	5743	0	5438	303	0	2
**1998**	5680	0	5475	203	0	2
**1999**	5635	160	4930	379	0	166
**2000**	5391	259	4794	284	0	54
**2001**	4432	305	3981	99	0	47
**2002**	2834	408	2191	234	0	1
**2003**	2948	798	1823	324	0	3
**2004**	2781	625	1747	387	0	22
**2005**	2673	661	1347	80	358	227
**2006**	1092	401	576	9	30	76
**2007**	996	280	697	1	0	18
**2008**	885	409	476	0	0	0
**2009**	93	69	24	0	0	0
**2010**	87	49	38	0	0	0

**All**	45907	4424	37669	2807	388	619

AS+AQ = artesunate+amodiaquine; Q = quinine; CQ = chloroquine; AQ+SP = amodiaquine + sulphadoxine/pyrimethamine. The other treatments were: SP, AQ, artemether-lumefantrine, AS-SP, AS or AS-mefloquine.

The policy of treating parasitologically confirmed cases with artesunate-amodiaquine was applied in 4,424 cases (44% 4,424/10,147 of the parasitologically-confirmed treatments). The annual number and proportions of AS+AQ treatments of the total consultations and treatment are presented in Figure 3.

From the clinical registries and the malaria cases projections it can be derived that during the 15 years under study 27,379 malaria treatments dispensed (or ~60% of all treatments) were not supported by a positive parasitological test (test not done or negative).

### Age-dependent risk of malaria during 1996-2010

**Figure 4:**
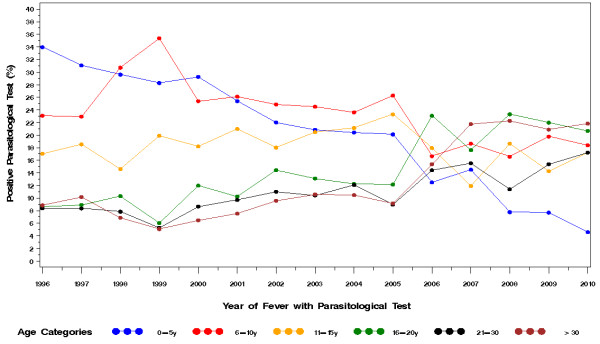
**Proportion parasitologically-positive malaria cases by age classes per year over the period 1996-2009**.

Figure 4 shows the proportion of parasitologically-positive cases of fever by age-group in 1996-2010. The age group 0-5y declined steadily throughout the entire study period; the 6-10y declined slightly; the 11-15y remained stable; the 16-20y, 21-30y and > 30y all increased.

**Figure 5:**
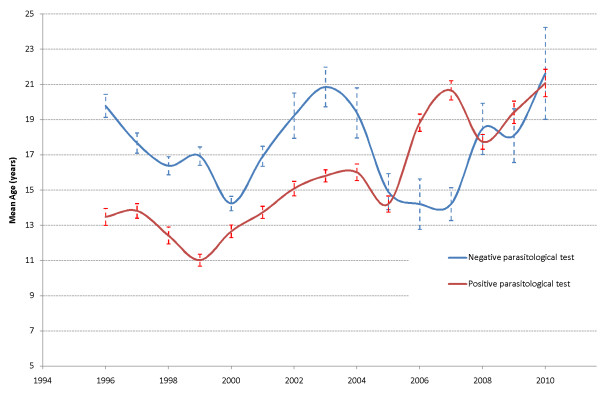
**Mean age of subjects with negative and positive parasitological tests during 1996-2009**.

The mean age of the malaria-positive patients increased steadily from 13.5 years in 1996 to 21.1 in 2010, while it fluctuated over time for the negatives, with similar values in 2010 (23.5 years) and 1996 (19.8 years) (Figure 5). The general linear model used to explain age by the time period under study and the parasitological test results showed a significant difference in the evolution of age during 1996-2010 between the malaria-positive and -negative subjects (p < 0.001 for year, tests and interaction).

### Parasitological test count model

**Figure 6:**
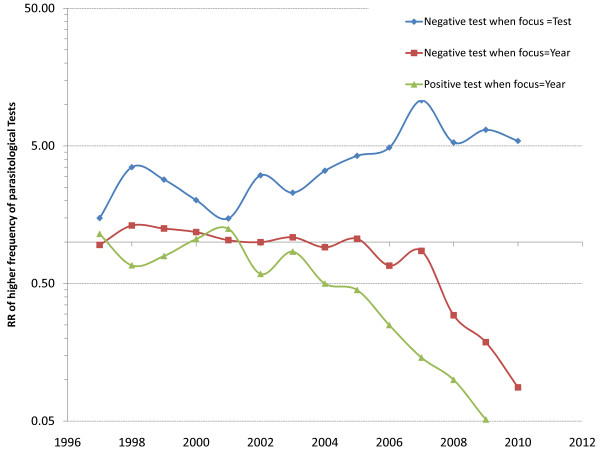
**Relative Risk (RR) of a positive or negative parasitological test by using the positive test as reference (blue line) or the year 1996 (red and green lines for negative and positive tests respectively) in the negative binomial model of counts of parasitological tests**.

From the negative binomial model of the count of parasitological tests, the interaction between test result and age showed that in 1996 all age categories had a lower risk of being tested for malaria (irrespective of the result) than the 0-5y. The interaction between test results and year showed that, overall, the risk of having a negative test was higher than that of a positive test, at all times (blue line in Figure 6). Compared to the risk of a positive test in 1996, the risk of a positive test (green line in Figure 6) became consistently lower (upper 95%CI of the RR < 1) starting from 2004, while this happened starting in 2008 for a negative test (red line in Figure 6).

When adding the interaction between year and age to the model, the results obtained for each age category over the years (Table 3) were similar to the above-described overall trends.

**Table 3:** Relative risk (RR) of positive malaria test by age classes during the period of 1997-2010 with respect to 1996 using a negative binomial model

	0-5y		6-10y		11-15y		16-20y		21-30y		> 30y	
**Year**	**RR**	**95%CI**	**RR**	**95%CI**	**RR**	**95%CI**	**RR**	**95%CI**	**RR**	**95%CI**	**RR**	**95%CI**

**1997**	1.15	[0.91; 1.45]	1.35	[0.77; 2.35]	1.25	[0.71; 2.20]	1.35	[0.75; 2.44]	1.33	[0.74; 2.38]	1.13	[0.64; 1.99]
**1998**	0.68	[0.53; 0.86]	0.92	[0.52; 1.62]	0.65	[0.37; 1.17]	0.77	[0.42; 1.41]	0.62	[0.34; 1.13]	0.53	[0.29; 0.94]
**1999**	0.79	[0.62; 1.01]	1.28	[0.73; 2.24]	0.90	[0.51; 1.58]	0.80	[0.44; 1.47]	0.70	[0.39; 1.27]	0.60	[0.34; 1.08]
**2000**	1.05	[0.83; 1.33]	1.27	[0.72; 2.21]	0.99	[0.56; 1.74]	1.77	[0.99; 3.17]	1.38	[0.78; 2.47]	0.56	[0.31; 1.00]
**2001**	1.25	[0.98; 1.59]	1.44	[0.82; 2.52]	1.23	[0.70; 2.17]	1.36	[0.75; 2.46]	1.24	[0.69; 2.23]	1.13	[0.64; 2.00]
**2002**	0.59	[0.46; 0.75]	0.77	[0.43; 1.37]	0.69	[0.39; 1.24]	0.94	[0.51; 1.71]	0.76	[0.42; 1.39]	0.64	[0.36; 1.16]
**2003**	0.85	[0.67; 1.08]	1.16	[0.66; 2.03]	1.11	[0.63; 1.97]	1.45	[0.81; 2.62]	1.30	[0.72; 2.32]	1.21	[0.68; 2.14]
**2004**	0.50	[0.39; 0.64]	0.58	[0.32; 1.04]	0.55	[0.30; 0.99]	0.67	[0.36; 1.25]	0.66	[0.36; 1.21]	0.60	[0.33; 1.09]
**2005**	0.45	[0.35; 0.58]	0.57	[0.32; 1.02]	0.51	[0.28; 0.92]	0.70	[0.38; 1.30]	0.45	[0.24; 0.83]	0.40	[0.22; 0.73]
**2006**	0.25	[0.19; 0.33]	0.25	[0.14; 0.48]	0.26	[0.14; 0.50]	0.62	[0.32; 1.18]	0.38	[0.20; 0.73]	0.40	[0.21; 0.75]
**2007**	0.14	[0.11; 0.19]	0.22	[0.12; 0.42]	0.13	[0.07; 0.26]	0.31	[0.16; 0.61]	0.27	[0.14; 0.53]	0.23	[0.12; 0.45]
**2008**	0.10	[0.07; 0.14]	0.19	[0.10; 0.39]	0.16	[0.08; 0.32]	0.32	[0.16; 0.67]	0.26	[0.13; 0.53]	0.33	[0.17; 0.66]
**2009**	0.05	[0.04; 0.07]	0.10	[0.04; 0.22]	0.09	[0.04; 0.19]	0.14	[0.06; 0.33]	0.11	[0.05; 0.26]	0.13	[0.06; 0.28]
**2010**	0.03	[0.02; 0.04]	0.09	[0.04; 0.23]	0.06	[0.02; 0.17]	0.17	[0.07; 0.43]	0.14	[0.06; 0.36]	0.15	[0.06; 0.36]

**Figure 7:**
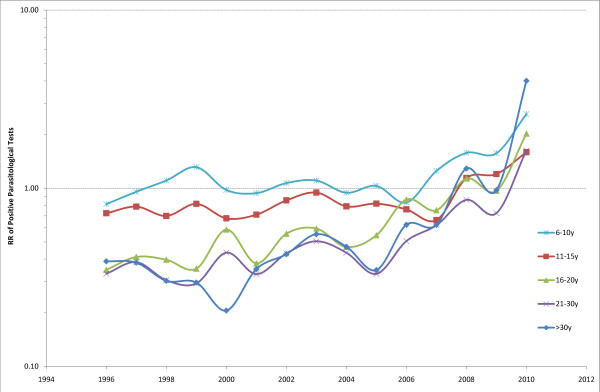
**Relative Risk (RR) for a positive parasitological test by using the 0-5y age category as reference in the negative binomial model of counts of parasitological tests**.

Figure 7 is based on the results of the interaction between year and age, and shows the RR of a positive parasitological test for each year of the period 1996-2010 for the different age categories vs. the age group 0-5y. The frequency of parasitological tests showed overall a similar trend for the 6-10y and 11-15y age categories over the years with occasionally significant RRs (Table 4) around the identity value (= 1) with a small increase in the period 2008-2010. The other three age categories showed a constant increase from 2001 to reach RRs > 1 for the 16-20y and > 30y age categories in 2010. Overall, these results show that, while in the past an age > 16 years had a lower risk for a positive malaria test, more recently the risk has become very similar across all ages.

**Table 4:** Relative risk (RR) of positive malaria test by age classes during the period of 1996-2010 with the age group 0-5y as the reference using a negative binomial model

	6-10y		11-15y		16-20y		21-30y		> 30y	
					
	RR	95%CI	RR	95%CI	RR	95%CI	RR	95%CI	RR	95%CI
**1996**	0.82	[0.64; 1.03]	0.72	[0.57; 0.92]	0.35	[0.27; 0.45]	0.33	[0.26; 0.43]	0.39	[0.30; 0.50]
**1997**	0.96	[0.55; 1.68]	0.79	[0.45; 1.40]	0.41	[0.22; 0.76]	0.38	[0.21; 0.70]	0.38	[0.21; 0.68]
**1998**	1.11	[0.63; 1.94]	0.70	[0.39; 1.25]	0.40	[0.21; 0.74]	0.30	[0.16; 0.56]	0.30	[0.17; 0.54]
**1999**	1.31	[0.75; 2.30]	0.82	[0.46; 1.45]	0.35	[0.19; 0.66]	0.29	[0.16; 0.54]	0.30	[0.16; 0.53]
**2000**	0.98	[0.56; 1.71]	0.68	[0.38; 1.20]	0.59	[0.32; 1.08]	0.44	[0.24; 0.79]	0.21	[0.11; 0.37]
**2001**	0.94	[0.54; 1.64]	0.71	[0.40; 1.26]	0.38	[0.20; 0.70]	0.33	[0.18; 0.60]	0.35	[0.20; 0.63]
**2002**	1.07	[0.61; 1.89]	0.86	[0.48; 1.53]	0.56	[0.30; 1.03]	0.43	[0.23; 0.79]	0.43	[0.24; 0.77]
**2003**	1.11	[0.63; 1.94]	0.95	[0.53; 1.67]	0.59	[0.32; 1.10]	0.50	[0.28; 0.92]	0.55	[0.31; 0.98]
**2004**	0.94	[0.53; 1.68]	0.79	[0.44; 1.42]	0.47	[0.25; 0.88]	0.43	[0.23; 0.81]	0.47	[0.26; 0.85]
**2005**	1.03	[0.58; 1.83]	0.82	[0.46; 1.47]	0.55	[0.29; 1.02]	0.33	[0.18; 0.62]	0.35	[0.19; 0.63]
**2006**	0.83	[0.46; 1.51]	0.76	[0.41; 1.40]	0.86	[0.46; 1.63]	0.51	[0.27; 0.96]	0.62	[0.34; 1.14]
**2007**	1.25	[0.69; 2.26]	0.66	[0.36; 1.23]	0.75	[0.39; 1.44]	0.62	[0.33; 1.17]	0.62	[0.34; 1.16]
**2008**	1.58	[0.85; 2.95]	1.14	[0.60; 2.19]	1.13	[0.58; 2.24]	0.86	[0.44; 1.68]	1.29	[0.69; 2.42]
**2009**	1.57	[0.80; 3.08]	1.20	[0.60; 2.42]	0.97	[0.46; 2.03]	0.73	[0.35; 1.50]	0.98	[0.50; 1.91]
**2010**	2.61	[1.27; 5.39]	1.60	[0.74; 3.46]	2.03	[0.94; 4.39]	1.63	[0.77; 3.48]	1.96	[0.96; 4.01]

### Malaria risk model

The logistic model of the risk of a positive parasitological test included the age of the subjects and the year of examination. The interaction was significant in the model. The models estimated the OR of positive parasitological test between 1-35 years of age by 1-year increments, and then to 70 years by five-year increments. This allows an analysis of the pattern of ORs change with increasing age.

The models showed a significant age effect from 1996 to 2003. The ORs were all decreasing regularly from 1y to 70y expressing a decreasing risk of positive parasitological test with increasing age. Between 2004 and 2010 with the exception of the year 2007 (risk increasing with age), age was no longer significant in the models. Overall, these results concur to show that in the past few years, the risk of malaria has become the same throughout life.

## Discussion

This study is based on over 45,000 cases of suspected malaria over 15 years at a rural dispensary in an area where malaria used to be mesoendemic and of moderate transmission intensity. Results document the decreased burden of malaria and fevers between 1996-2010 and the concurrent increase in the age of malaria patients. Together, these variations indicate reduced malaria transmission.

The 32-fold drop in the calculated risk of malaria was paralleled by changes in the age profile of malaria patients, so that the risk of malaria is now almost uniformly distributed throughout life, while in the past malaria used to concern more children under 16 years of age.

The age-dependent risk of malaria and its clinical presentation (uncomplicated and or severe) varies with transmission intensity. Marked differences in the number of malaria attacks at different ages occur in varying epidemiological settings (reviewed in Lalloo *et al *[17]). Obviously, intensity of transmission and number of challenges over time determine the susceptibility to malaria infection and disease, as a function of building immunity. The age profiles of malaria cases vary greatly even across areas of stable malaria, depending on the intensity of transmission [16].

While trends towards decreased malaria transmission, morbidity and mortality are being reported world-wide [3-5,8], very little information exists on how these changes affect different ages. In 2008, O'Meara *et al *[4] reported that while the age of non-malaria fevers seen at a paediatric word in Kilifi, Kenya has remained unchanged between 1990-2007 (2.15 to 2.18 years), that of slide-positive patients increased from 2.3 to 3.6 years. In Mlomp, the mean age of the malaria patients increased steadily from 13.5 years in 1996 to 21.1 in 2010, while non-malaria fevers remained stable (19.8 years in 2007 and 23.5 in 2010). While overall in agreement with the trends presented by O'Meara *et al *[4], the situation here is clearly different from coastal Kenya where intensity of transmission is higher, age of patients lower, and where the curves of the malaria-positive and malaria-negative hospital admissions started diverging already in the 1990s.

The challenge with these data was to translate an observation into a statistically robust analysis of trends over time, hence the use of models, which allow the study of interactions between variables. All these analyses converge to show a progressive shift in the age-dependent risk of malaria, with increasing risk for adolescents and adults (16 years and older) with decreasing malaria prevalence.

Between 1996 and 2010 significant changes have been introduced to malaria policies and practices here like elsewhere. In Senegal, the official policy change from chloroquine or quinine given on clinical grounds to artesunate-amodiaquine on parasitological confirmation was made in 2006, but RDTs were made available from 2007. In Mlomp, parasitological diagnosis with microscopy was applied occasionally in the 1990s and then more systematically from 2000 when piloting the implementation of the new recommendations of the World Health Organization [18] to use artemisinin-based combinations for parasitologically-confirmed malaria. In this sense, Mlomp may be different from other settings in the country and the changes seen here not be obvious elsewhere, at least as yet. However, they indicate what changes may be expected in other settings which have been implementing these measures for a shorter period of time. The decreased parasite-positive rates cannot be ascribed to different performance of microscopy and RDTs as this is regularly tested locally and no significant discrepancies are found.

**Figure 8:**
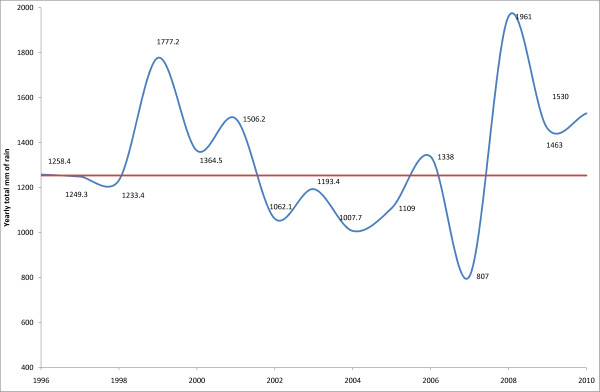
**Average yearly rain falls in Mlomp for the period 1996-2010**.

A temporal association between the reduced malaria risk and the availability of an effective treatment (artesunate-amodiaquine) is apparent but there is no decisive evidence of a causal relationship. Rainfalls cannot explain the changes: they fluctuated overtime, but have not decreased - if anything, there has been more precipitations in 2008-2010 than in 1996-1998 (Figure 8). Distribution, use and effects of bed nets are difficult to quantify. Data for the entire country [19] report an increase between 2005-2010 in the number of households with an impregnated bed net from 20% to 82%. In 2010, 45% of children under 5 and 35% of the 5-14 year olds slept under a bed net, as compared to 25% and 31% of men aged 15-49 and adults over 50. More specifically for Mlomp, records available at the district level show that a total of 1,518 bed nets were distributed in the period 2002-07 (308 in 2002, 200 in 2003, 165 in 2004, 500 in 2005, 60 in 2006 and 285 in 2007) for a population of ~7,600. This does not include other providers (e.g. non-governmental organizations). A local study (unpublished) found in 2010 that 48% of the households interviewed utilise bed nets correctly. The RBM report [19], local experience and other data [20] concur that suboptimal use of bed nets occurs when the "perceived benefits of reduction in mosquito nuisance and [the risk] of malaria [are] considered not to be worth the inconvenience of daily use". While local data are not available, the country data on lower utilisation of bed nets by adults might contribute to the relative increase in risk of malaria observed in this setting.

Changes in users' and health providers' behaviours may be possible. Attendance to the health facility has decreased steadily overtime and consultations now are ~40-50% of those in the 1990's. It may be argued that people are turning away from the dispensary. However, this seems not to be the case as there is no private sector in the village and attendance to other facilities of the district has not increased. Therefore, this may be a result of less illness in the community. The number of consultations which were not treated as malaria (on any grounds) dropped and was in 2002-2006 circa half of those seen in the 1990s, but started raising again and is ~80% in 2007-2010.

Comparing local data to the official data from the national malaria control programme for the country is not easy; country malaria statistics included in the past both clinical and parasitological diagnoses; the wide-scale implementation of parasitological diagnosis with RDTs started in 2007. Mean malaria morbidity for the country was stable between 2001 and 2006 (36%-34%) and then dropped steadily to reach 7% in 2008 and 4% in 2009. The total number of cases reported to the national programme dropped from 1,555,310 in 2006 (clinical diagnosis) to 170,890 in 2009 (parasitological diagnosis) (Ministère de la Santé et de la Prévention, République du Sénégal 2010).

The total number of cases reported to the national programme dropped from 1,550,000 in 2006 and 1,170,00 in 2007 (malaria suspected cases) to 295,000 in 2008 and 174,000 in 2009 (confirmed malaria cases) [19]. A recent paper on national trends between 2007-09 [21] also reports a drop in both the number of suspected and confirmed cases, as well as the number of treatments, after introducing RDTs country-wide. Of note, on average throughout the country 86% of the suspected cases were tested in 2009 and malaria infection was detected in ~30%.

In summary, this study shows that, in this setting originally with moderate malaria transmission, the risk of malaria has decreased over time, with a redistribution of the risk across ages, and that a similar change is not observed for the non-malaria fevers. It is legitimate to infer that less malaria is driving improvements in general health in this community; when comparing the last four years (2007-2010) to pre-2000 years, the drop in malaria (estimated cases) was > 96%, which is comparatively much larger than the decrease in non-malaria fevers (estimated) which dropped by 46% (54% for total consultations for fever). Furthermore, effects of reduced malaria and the possible interaction between malaria and other febrile illnesses, especially in children, may translate into long-term benefits that are difficult to quantify with the information available at this stage. Finally, over-treatment of malaria decreased with the new policy.

## Competing interests

The authors declare that they have no competing interests.

## Authors' contributions

PB was the Principal Investigator of the study. He contributed to the concept, protocol, analysis and reporting of the study, and contributed to the preparation of the manuscript. He personally contributed to the treatment, follow-up of patients and quality control of the study. MC and MB participated in the planning and supervised the implementation of the study. PA contributed to the treatment and follow-up of patients. MV designed and conducted the analyses, and prepared the manuscript. PO contributed to the concept of the project, design of the protocol and analyses, reporting of the study, and prepared the manuscript. All authors read and approved the final manuscript.

## Disclaimer

PO is a staff member of the WHO; MV is a staff member of the CRP-Santé; the authors alone are responsible for the views expressed in this publication and they do not necessarily represent the decisions, policy or views of the WHO or the CRP-Santé.
